# Prevalence of disordered eating in athletes categorized by emphasis on leanness and activity type – a systematic review

**DOI:** 10.1186/s40337-020-00323-2

**Published:** 2020-09-29

**Authors:** Ryley P. Mancine, Donald W. Gusfa, Ali Moshrefi, Samantha F. Kennedy

**Affiliations:** 1 Medical Student, Michigan State University College of Osteopathic Medicine, Michigan State University, East Lansing, MI 48825 USA; 2 Lab Personnel, Michigan State University College of Osteopathic Medicine, Michigan State University, East Lansing, MI 48825 USA; 3 Child and Adolescent Psychiatrist, Department of Psychiatry, Michigan State University, East Lansing, MI 48825 USA

**Keywords:** Eating disorders, Sport group, Sports mental health, Leanness, Activity group, Disordered eating

## Abstract

**Background:**

Disordered Eating (DE) shows a strong association with athletics and can lead to several negative mental and physical health effects. Traditionally, sports have been grouped based upon whether or not the sport emphasizes leanness as a competing factor. Due to sociocultural factors, risk for DE may also be associated with the sport type. The aim of this review is to critically analyze the available research and data in this field to consider the relationship between DE and sport type to see which factors influence prevalence among athletes

**Method:**

A systematic review was completed using keywords specific to DE and sport types. Articles were either excluded due to lack of specification of athlete type or failure to use a standardized screening tool or interview for data collection.

**Results:**

6 out of 7 studies found a significant increase in DE rates among lean sport types. When classifying by sport type reports were less consistent, but show non-lean sports also have increased rates of DE.

**Conclusion:**

There are variations in prevalence of DE behaviors depending on athlete type. It is important to identify the risk for DE early in athletes so emphasis can be placed on treatment options to nullify progression to an eating disorder, lower negative impacts on an athlete’s performance, and prevent other negative health effects. Using sport groups is important to clinical practice as well as research, as certain sports may have a higher risk for development of DE.

## Plain english summary

For athletes, disordered eating can progress to an eating disorder, which has a multitude of physical and mental health consequences. Certain types of athletes may be at an increased risk for disordered eating behaviors. This manuscript attempts to categorize sport types to determine which groups of athletes may have the highest incidence of disordered eating. It also attempts to determine if there are major differences in the presentation of disordered eating behaviors between different sport types. It is important to identify the risk for disordered eating in athletes of all types so that disordered eating behaviors may be halted before they progress to an eating disorder, which is much more difficult to treat. This systematic review of the literature aims to discover differences between the different types of sport as they relate to disordered eating behavior rates, risks, and pathologies.

## Introduction

Over the past fifteen years, there has been an increase in research on eating pathology in sports [1]. Eating pathology is often described as a continuum ranging from disordered eating (DE) to a clinical eating disorder (ED). DE encompasses symptoms of dysfunctional eating patterns such as fasting, dieting, vomiting, over-eating, binge eating and use of laxatives and/or diet pills [2]. In athletes, DE frequently occurs due to the desire to achieve a sport-specific body-ideal and alleviate sport-specific body dissatisfaction [3]. DE can lead to EDs if left unaddressed and can cause increased incidence of mood, anxiety, and substance abuse disorders [4, 5]. ED are clinical diagnoses that meet DSM-5 criteria, including Anorexia Nervosa (AN), Bulimia Nervosa (BN), and Other Specified Feeding and Eating Disorder (OSFED) [6, 7].

Estimates of the prevalence of DE among athletes varies widely in research due to the different populations of athletes studied (different sports, ages, levels of competitiveness, gender, etc.). In a study of DE amongst elite adolescent athletes by Martinsen et al., 606 Norwegian first year elite sport high school athletes reported higher prevalence of DE compared to the control group [8]. It was found that in the athlete group there was a significantly higher prevalence of DE in females who participated in lean sports compared to males in the same group [8]. Athletes competing in sports such as gymnastics, figure skating, diving, and dancing, where leanness is emphasized, have been found to be at higher risk of DE, which frequently leads to a decrease in sport performance [9].

Perfectionism plays a role in the psychological impact of DE in an athlete, acting both as a symptom and a risk factor for DE [10]. Perfectionism often influences an athlete to have unrealistic expectations, which can result in dissatisfaction with body image and sport performance [5]. Research has found a correlation between DE and perfectionism, with an emphasis on precision and personal expectation for an athlete to achieve a sport-specific body to improve performance [10].

The female athlete triad encompasses three disorders in female athletes: DE, amenorrhea and osteoporosis [11]. This is demonstrated in the desire to lose weight to achieve a sport-specific body ideal, commonly resulting in an energy deficit, which may lead to amenorrhea and osteoporosis. Additionally, this energy deficit will ultimately result in poor performance [10]. A study by Cobb et al. examined 91 competitive female distance runners ages 18–26 years and found that female runners with poor nutrition and irregular eating patterns had an energy imbalance, which often led to amenorrhea [12]. The female athlete triad refers to female athletes of all kinds of sports, regardless of sport type category. While the term ‘female athlete triad’ has been established for many years, more recently the term ‘Relative Energy Deficiency in Sport’ (RED-S) has been used to describe these same three traits coupled with a multitude of other systemic consequences that are associated with low energy availability [13]. RED-S syndrome demonstrates the multitude of physiological consequences associated with DE, such as hematological, cardiovascular, and gastrointestinal disruptions.

**Fig. 1:**
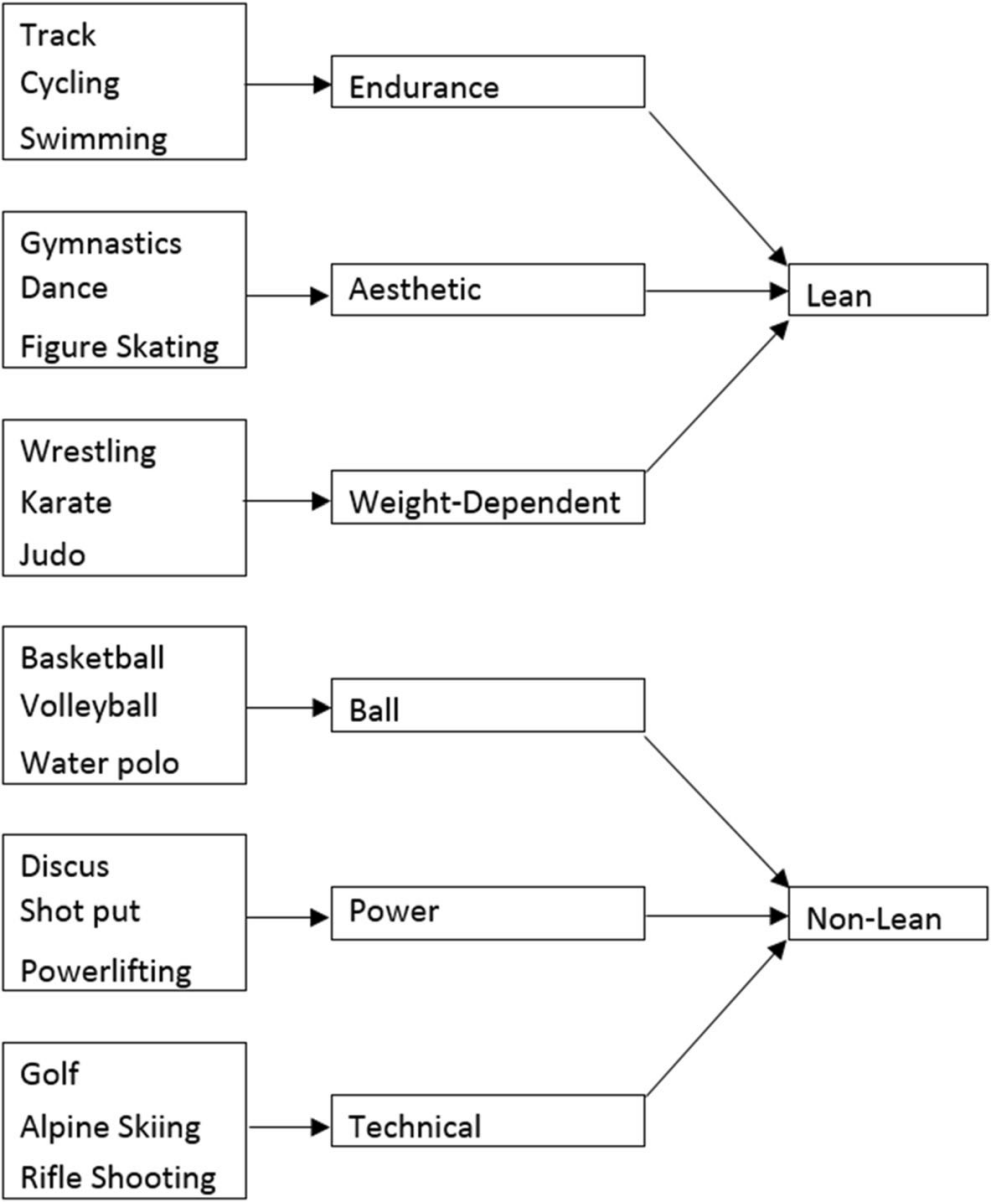
Examples of various sports and the categories they fit in. The groupings of sports that define lean and non-lean sports are also provided

Athletes can be divided into lean and non-lean categories. Lean sports emphasize achieving and maintaining a lower body weight due to the belief that lower body weight improves performance [8]. A few examples of lean sports include dancing, judo, long-distance running, swimming, and diving [8, 14]. Alternatively, non-lean sports do not require a low body weight in order for an athlete to be competitive [15]. Some non-lean sports include golf, basketball, table tennis, and horse riding [8]. Lean sports may increase risk for DE because athletes may engage in pathogenic weight-control behavior to achieve a lower body weight [16]. A study by Kong et al. found athletes participating in lean sports scored higher on the Eating Attitudes Test (EAT-26) compared to athletes competing in non-lean sports. Additionally, lean sport athletes reported significantly more eating pathology compared to non-competitive athletes with 84% of the female athletes who screened positive participating in lean sports [15, 17]. Examples of sports that fit into each category, along with categories of sports that make up lean and non-lean sports, are referenced in Fig. 1.

Sports can be further divided into six categories: aesthetic, weight-dependent, endurance, ball game, power, and technical sports [18]. Of these categories, aesthetic, weight-dependent and endurance sports are typically considered lean sports, whereas ball game, power and technical sports are considered non-lean sports.

In aesthetic sports, the performance of an individual or team is assessed by a judge or judges of the competition [19]. In aesthetic sports, the winner is determined by judging an individual or team performance using a complex set of rules [19]. Thus, appearance is a major factor in the judging. Examples of aesthetic sports include gymnastics, diving, figure skating, dancing, ballet [16]. All of these sports are considered lean sports due to the pervasive belief that a lower body weight results in more favorable judging [20].

Weight-dependent sports divide competitors into different categories based on the weight of the competitor. Examples of weight-dependent sports include wrestling, karate, and judo [18]. Studies have shown athletes competing in weight-dependent sports are at an increased risk for eating pathology, such as DE, compared to nonathletes [21]. This is likely due to athletes attempting to achieve lower body weight while maintaining muscle mass to gain a competitive advantage by competing in a lower weight class. Athletes competing in weight-dependent sports may utilize pathogenic weight control behaviors to achieve rapid weight loss prior to a competition in order to achieve higher levels of success [8]. Due to this emphasis on achieving a low body weight, weight-dependent sports can be categorized as lean sports.

Endurance sports include cycling, rowing, running, swimming, cross-country skiing and speed skating [18, 22]. In these sports, a lower body weight is typically associated with a higher level of competition [23]. As a result, endurance athletes may utilize abnormal eating behaviors to achieve a body weight that is too low, resulting in an energy imbalance. This imbalance of energy is a frequent consequence of DE in endurance athletes due to the high metabolic requirements of high-volume aerobic training [22, 24]. Thus, endurance sports can also be categorized as lean sports.

Ball-game sports involve a ball where the objective is to move the ball between members of the same team with a specific goal to score more points than the other team [18]. Ball sports include football, soccer, and volleyball, as well as bat/stick sports (hockey, baseball, cricket) [15, 18]. Ball-game sports are considered non-lean sports as performance is determined by the athlete’s ability to maneuver the ball as desired and thus is not dependent on a specific weight.

Power sports emphasize strength and include powerlifting, shot put, and sprinting [18, 25]. The goal in these sports is to maximize strength and power to improve performance [25]. Appropriate nutrition plays a fundamental role in the athlete’s ability to increase strength and muscle mass for competitive success [25]. Thus, power sports are considered non-lean sports due to this desire to achieve strength.

Technical sports place an emphasis on a certain skill with a piece of specialized equipment [18]. An example of a technical sport is rifle shooting [18]. There has been very little research on eating behaviors in technical sports. However, as this category of sport emphasizes a specific skill and not a specific body type, it can be categorized as a non-lean sport.

Some sports may have higher prevalence rates of DE than other sports, and it is important to consider different activities and focus groups when assessing for prevalence of DE. Athletes who participate in endurance sports have a higher emphasis on aerobic training compared to others [18]. When emphasis is placed on different aspects of training and competition, one can expect an outcome that entices an athlete to continue pursuing the method that allowed them to gain a competitive advantage. In doing so, this may result in the athlete normalizing irregular diet patterns/quantity with respect to their training, resulting in DE.

This systematic literature review is, as far as the authors are aware, first of its kind to date to examine current data in the field of DE in leanness athletes and other activity types to assess for prevalence. It is critical to understand the potential risk factors that are present for athletes, making them susceptible to further health complications. By evaluating the athlete for DE, it facilitates early detection of irregular eating patterns that could lead to ED. This systematic review aims to address the variable prevalence and presentation of DE behaviors in the various types of athletes.

## Methods

### Search strategy and data reporting

The PubMed database was the primary source of collected articles. Articles accessed were published between January 2000 and July 2019. All articles were obtained after the year 2000 to provide an insight into the most recent developments of research within the field. Articles were screened for the terms “Athletes,” “Disordered Eating,” “aesthetic,” “power,” “endurance,” “technical,” “ball,” “weight dependent,” and “leanness.” Articles that did not directly answer the research question “Which athlete groupings would be more susceptible to DE?” or report on DE or athletes specifically were not included. This review article follows all acceptable research practices and ethical considerations as outlined by Navalta et al. [26]. Suitable articles in this study were individually reported on, with correlation and possible weaknesses being stated in the interpretation portion of the tables. These interpretations were then reviewed by our research team to validate the original analysis. All participants defined as athletes (novice or elite) were included in this review to allow for significant amounts of data to be collected. These athletes needed to be defined as having DE using at least one standardized screening tool or interview. There were no restrictions on control groups.

### Selection of studies

**Fig. 2:**
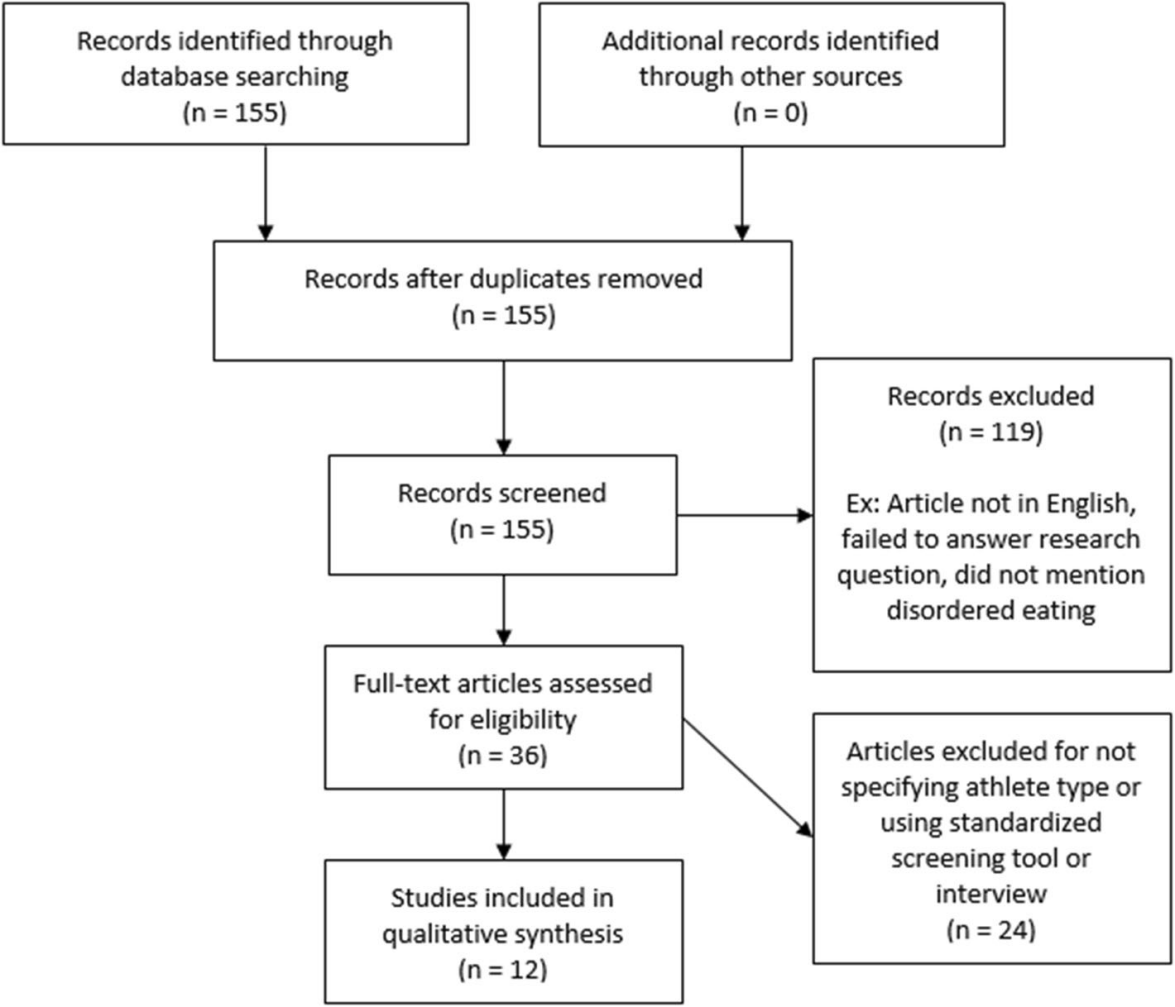
PRISMA-guided literature search methods

A detailed flowchart of the search strategy can be seen in Fig. 2. 155 articles were initially identified in a preliminary literature search, but initial text analysis for keywords resulted in 119 articles being removed from analysis. Articles were included based on the following analysis of abstracts: [1] peer-reviewed published work in the English language, [2] specific mention of DE or an ED in athletes, [3] outcome measures which included prevalence rates of DE. Each included article was reviewed independently by two authors to ensure appropriate criteria were met. Evaluation of the subsequent texts based on relevance to the hypothesis resulted in the removal of 9 articles, leaving 27 in the review. Further articles were excluded upon full-text analysis due to their lack of specification of athlete type or that they did not use a standardized screening tool or interview such as the EAT-26 or EDE-17. Therefore, there were 12 articles that specifically fit the research question. These athlete types were reported in two tables based on whether they were categorized as “lean” or a specific sport type. If an individual sport was reported it was categorized into the prior mentioned categories.

## Results

### Comparison of lean vs non-lean sport groupings

Research on lean sports found athletes participating in lean sports had significantly higher rates of DE than non-lean sports in 6 out of 7 studies (Table 1). In one of these studies, different sports did not have a statistically significant difference in rates of DE [29]. However, when the sports were grouped into “Lean” and “Non-Lean” categories, the difference in the rates of DE between the two categories was statistically significant.

**Table 1:** Comparison of DE prevalence between Lean vs Non-Lean sports

***Author***	***Population***	***Sport Grouping***	***Measures/Analysis***	***Results***	***Interpretation***
Rousselet et al., 2017 [27]	High level French athletes over the age of 12 (*n* = 340)	Lean vs Non-lean	Positive result was measured as one or more positive assessment after three consecutive clinical interviews performed by healthcare professionals. Multivariate analysis used to detect difference between groups.	Lean sport athletes had a significantly higher prevalence of clinician-detected DE (*p* < 0.01)	Strong correlation between lean sports and increased prevalence of DE
Wells et al., 2015 [28]	United States Division I female college athletes (*n* = 83)	Lean Sports: cheerleading, cross country/track, swimming, volleyball Non-lean Sports: basketball, softball, swimming, golf	Athletes were administered the ATHLETE questionnaire. Results were then analyzed using one-way ANOVA.	No statistically significant difference between individual sports, but when grouped into Lean vs No-Lean, lean sports had a higher prevalence of DE	Positive correlation between lean sports and increased prevalence of DE
Martinsen et al., 2010 [8]	Norwegian first year elite sport high school athletes (*n* = 606) and control high schools students (*n* = 355)	Lean vs Non-lean	Athletes were administered a questionnaire measuring subscales from the Eating Disorders Inventory (EDI). Leanness results detected using regression analysis.	Higher prevalence of DE was found in female athletes of lean sports when compared to boys in lean sports *p* < 0.001, but not across sport groups	Lean sports correlated with higher prevalence of DE, however, the author included a wide range of non-traditional sports classified as a lean sport which may not have been an appropriate fit
Torstveit et al., 2008 [3]	National athletes of junior or senior level (*n* = 186) and a control sample (*n* = 145)	Lean vs Non-lean	Athletes screened positive if had a positive screen using the EDI-DT or EDI-BD. Results detected using t-test and X^2^ analysis.	Lean athletes (47.7% positive) were found to have significantly higher prevalence of DE than Non-lean athletes (19.8% positive) and controls (21.4% positive) *p <* 0.001	Lean sports strongly correlated with higher prevalence of DE
Vardar et al., 2007 [29]	Turkish female athletes with a mean age of 19.59 (*n* = 243)	Lean (*n* = 72) vs Non-lean (168)	Athletes were administered the EAT-40 questionnaire. Results were then analyzed using t-test and X^2^ analysis.	No statistically significant difference found in prevalence of DE in lean vs non-lean sports	No correlation between lean sports and higher prevalence of DE
Rosendahl et al., 2009 [30]	German athletes (*n* = 576) and non-athletes aged 14–18 (*n* = 291)	Lean (*n* = 228) vs Non-lean (*n* = 245) Endurance, Aesthetic, Weight dependent, Antigravitation, Technical, Ball Game, Power	Athletes were administered the EAT-26 questionnaire. Results were then analyzed using logistic regression, unpaired t-test and X^2^ analysis.	Lean sports had a higher prevalence of DE in males and no significant difference in females. The only sport types with significant increase in prevalence of DE were “Antigravitation” when comparing males and “Power” when comparing females.	Lean sports correlated with higher prevalence of DE in males
Kong et al., 2015 [15]	Australian female athletes aged 17 to 30 years regularly participating in sports. (*n* = 320)	Lean (*n* = 174) vs Non-lean (146)	Athletes were administered the EAT-26 questionnaire. Results were then analyzed using ANOVA.	Lean athletes scored higher on the EAT-26 than non-lean athletes	Higher prevalence of DE among lean athletes Of note, 60.9% of the lean group competed in aesthetic sports, thus those athletes could have a larger effect on the sample

### Comparison of activity type sport groupings

Five studies divided sports into activity subgroups, which were then analyzed for risk for DE (Table 2). Two studies found that aesthetic sports had a higher prevalence of DE [6, 9]. Aesthetic sports place an emphasis on leanness and thus are considered lean sports [20]. This could explain the lack of a statistically significant difference in prevalence rates of DE in the study by Vander et al. as only 6 of the 72 athletes studied competed in aesthetic sports [29]. By contrast, in the study by Kong et al., 60.9% of the lean athletes competed in aesthetic sports [15]. This study found a higher prevalence of DE in lean sports.

**Table 2:** Comparison of DE prevalence in athletes of sports classified by activity type. “+” indicates activity type defined as lean

***Author***	***Population***	***Sport Grouping***	***Measures/Analysis***	***Results***	***Interpretation***
Krentz et al., 2013 [6]	Adolescent athletes from elite sports schools and Olympic training centers (*n* = 65)	+Aesthetic: gymnastics, ice/roller figure skating, ballet, and rhythmic gymnastics	Athletes were administered the Emotional Element of Exercise questionnaire. Results were then analyzed using ANOVA.	DE in males was measured to significantly decrease over time of one year, while females remained constant	Aesthetic sports have higher prevalence of DE in females versus males
Krentz et al., 2011 [9]	Elite athletes (*n* = 96) vs Control (*n =* 96) with a mean age of 14.0 years	+Aesthetic vs Control	Cross-sectional study where athletes were administered the EAT-26. Results were then analyzed using ANOVA.	Aesthetic athletes had higher prevalence of DE vs control (*p* < 0.03); female athletes had higher prevalence of DE than male athletes (*p* < 0.001)	Aesthetic sports had higher prevalence of DE; females had higher prevalence of DE than males, but both had similar rate of increased prevalence when compared to a control
Chatterton et al., 2013 [31]	United States male college athletes of various levels, mean age 19.91 years (*n* = 732)	+Endurance, +weight-dependent, ball game	Athletes were administered the Questionnaire for Eating Disorder Diagnosis (Q-EDD). Results were then analyzed using X^2^ analysis.	Weight-dependent sports (44.2%) had higher prevalence of DE than Endurance (12.8%) and Ball Game (16.7%) sports (*p* < 0.001)	Strong correlation between male weight-dependent sports and higher prevalence of DE
Kampouri et al., 2019 [32]	Greek elite female athletes mean age of 23.10 (*n* = 129) and female non-athletes (*n* = 46)	Ball Sport vs Non-Athlete 53 Basketball 42 Volleyball 34 Water Polo 46 Non-athletes	Athletes were administered the Eating Disorders Questionnaire (EDE-Q). Results about inter-sport difference were analyzed using ANOVA.	Using the EC subscale Water Polo athletes had higher scores than basketball or volleyball (*p* < 0.05)	Water polo had higher prevalence of DE, while other ball sports did not.
Rosendahl et al., 2009 [30]	German athletes (*n* = 576) and non-athletes aged 14–18 (*n* = 291)	Lean (*n* = 228) vs Non-lean (*n* = 245) +Endurance, +Aesthetic, +Weight-Dependent, +Antigravitation, Technical, Ball Game, Power	Athletes were administered the EAT-26 questionnaire. Results were then analyzed using logistic regression, unpaired t-test and X^2^ analysis.	Sport types with significant increase in DE were “Antigravitation” when comparing males and “Power” when comparing females.	Power and Antigravitation sports correlate with higher prevalence of DE depending on gender

A study by Chatterton et al. analyzing DE in male athletes found weight class sports had a significantly higher prevalence of DE compared to endurance and ball game sports [31]. A study by Rosendahl et al. analyzed multiple sport types and found Power and Antigravitation sports had a significantly higher prevalence of DE when adjusted for gender. Additionally, they found Endurance, Aesthetic, Weight-Dependent, Technical and Ball Game sports had no statistically significant difference [30]. Kampouri et al. analyzed three Ball Game sports and found different prevalence rates of DE between the sports. They found water polo had a higher prevalence of DE than volleyball or basketball [32]. Currently, there is not enough information on which sport or sport type has relation to DE indicating further research is required for clarification on which sports have the highest risk for DE.

Lean sports have a higher prevalence of DE when studied either directly (Table 1) or indirectly (Table 2). However, there were multiple inconsistencies regarding the higher prevalence of DE in lean sports when specific sport types were studied (Table 2). For example, some non-lean power [30] sports were found to have a higher prevalence of DE while some endurance [30, 31] lean sports did not show statistically significant differences. This implies that sensitivity may be higher when dividing sports as lean versus non-lean [32], but specificity may be higher when dividing sports by type (Aesthetic, Weight-dependent, Endurance, Ball Game. Power, Technical) [30].

## Discussion

DE is common amongst athletes, but there is still much to learn about the prevalence within certain subgroups. Though there is clear evidence that an emphasis on leanness plays a role [3, 8, 15, 27, 29, 30], divisions into further subgroups show less concise evidence. When subdividing sports into activity type, aesthetic sports seem to be consistently correlated with an increase in DE rates [6, 9]. Further, the only article who found lean sports did not show higher rates of DE did not have many aesthetic sport athletes within their lean group [29]. This indicates that, though useful, the lean grouping does not provide a complete picture when addressing DE risk. The rest of the significant positive findings were shown in weight-dependent [31], power [32], antigravitation [32], and the specific ball sport water polo [27]. These provide insight into the risk of more specific groupings and therefore the risk factors of DE in athletes, but due to lack of evidence nothing more definitive can be said about the sport type groupings at this time. That said, these findings could serve as a guide for further research aimed at defining these rates within certain sports.

Athletes participating in lean sports compete in different environments and train differently than athletes participating in non-lean sports. Thus, risk factors for DE in athletes are unique to the demands of the sport environment (e.g. high-pressure situations with constant observation and performance-based evaluations). With knowledge of potential high-risk groups, physicians of multiple fields are better equipped to identify a patient with DE for treatment. In general practice this knowledge paired with the longitudinal relationship built with an athlete puts the primary care physician at a unique intersection to allow for diagnosis and treatment of DE, thus preventing progression to an ED, the female athlete triad and RED-S, and other nutritional problems. Sports medicine physicians and ancillary staff having similar if not greater amounts of contact with athletes when compared to general practitioners should share a similar role, identification, and management. Psychiatrists, though they may have a lower level of exposure in order to screen for athletes, should be very aware of these higher risk groups. Given the relative ease of treatment for patients with DE in comparison to EDs, a psychiatrist or other healthcare professional with a properly prepared treatment plan can prevent the progression of DE to a more severe ED.

This systematic review is limited by the small availability of research on DE or EDs on specific athletes. Additionally, this research is limited by the fact that only one database was searched for article collection and the fact that no grey literature (literature produced by academic or commercial sources which was made available outside of traditional publishing processes) was considered. Future research regarding athletic groupings should be focused on uncovering pathological differences in the presentation of DE in these various sport categories. Subsequent research on DE and EDs should pay close attention to weight trends in various types of athletes. Despite these weaknesses, this research is the first of its kind to categorize the prevalence of DE in athletes. DE patterns in Aesthetic athletes and Weight-dependent athletes should specifically be addressed as rates in those two sport types show evidence of being particularly high. Future screening tools which are developed for the assessment of DE or EDs in athletes should be validated in wide ranges of athlete types as prevalence rates and symptom expression may be variable among different types of sports. Developing tools or performing research in a single athletic population will severely limit the generalizability of that research, and comprehensive, multi-sport research should be done when referring to athletes as a group.

## Conclusions

Health professionals caring for athletes should be aware that there is strong evidence indicating athletes competing in lean sports are at higher risk for DE, especially Aesthetic athletes and male Weight-dependent athletes. However, as the risk in other sport types is not yet clear, all athletes should be screened for DE. All athletes should be provided education on proper nutrition and, if needed, resources for treatment of DE and EDs. It is critical to remember that not all athletes experience the same symptoms or symptom severity when performing research on DE or EDs in athletes or treating those pathologies as a physician. Although categorizations and groupings of athletes provide limited guidance when preparing an appropriate sample for data collection or treatment modality, a wide variety of athlete types provides a more representative example of an athletic population. Currently, there is not enough information to define specific sport populations based on risk for DE. If we are to properly address this problem, then further research needs to be completed with the aim of better identification of DE in athletes. As it currently stands, aesthetic athletes and the subgroups that compose it are the highest risk for disordered eating behavior.

## Data Availability

Not applicable – all citations provided.

## Abbreviations

DE: Disordered eating
ED: Eating disorder
AN: Anorexia nervosa
BN: Bulimia nervosa
EAT-26: Eating Attitudes Test
OSFED: Other specified feeding and eating disorder
RED-S: Relative Energy Deficiency in Sport
